# Joint function in marmosets and tamarins: Insights from computational modeling of hip extensor muscles

**DOI:** 10.1111/joa.14268

**Published:** 2025-05-15

**Authors:** Patricia Berles, Jan Wölfer, John. A. Nyakatura

**Affiliations:** ^1^ Comparative Zoology Institute of Biology, Humboldt‐Universität zu Berlin Berlin Germany

**Keywords:** locomotor ecology, muscle moment arms, muscle strain, primates

## Abstract

Analyses of the musculoskeletal system of callitrichid primates contribute to the understanding of the specializations of an apparently highly conserved body plan exhibited by this radiation of New World primates. This pilot study provides data from computational modeling of muscle function of five hip extensor muscles in four species of Callitrichidae to identify potential adaptations to previously documented differential leaping behaviors. Based on microCT scans of fresh cadavers, we reconstructed the muscle topology to inform the modeling of instantaneous muscle moment arms (MMAs) contributing to hip extension and accompanying muscle strains. Generally, muscle properties of the four species were surprisingly similar despite documented differences in leaping behavior. However, all extensors of Goeldi's marmoset (except for the semimembranosus) had the longest instantaneous MMAs. This may result in a greater capacity to generate hip torques in these marmosets (assuming identical force provided by the muscles), beneficial to their specialization in long‐distance trunk‐to‐trunk leaps. The shorter instantaneous MMAs of the extensors of the three other studied species indicate specialization toward more rapid hip extension. Strain analysis showed that, in all four species, the two glutei optimally generate force during the entire extension of the hip from a strongly crouched leg position to take off with an almost entirely extended leg. For the other three muscles (biceps femoris, semimembranosus and semitendinosus), we found optimal strains for force generation only at 50°–140° hip extension. We tentatively conclude that a relatively generalized musculoskeletal system for hip extension, coupled with moderate biomechanical adaptations favoring either joint torque or rotational speed, enables callitrichids to achieve remarkable locomotor versatility within highly intricate arboreal environments.

## Graphical abstract

This pilot study uses computational modeling to analyze five hip extensor muscles in four Callitrichidae species, aiming to identify adaptations to different leaping behaviors. Muscle topology was reconstructed from microCT scans to model instantaneous muscle moment arms and muscle strains during hip extension.

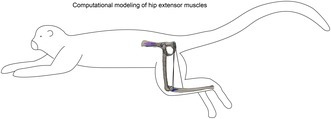

## 1 BACKGROUND

Callitrichidae (a group of New World primates comprising marmosets and tamarins) constitute an interesting study case for evolutionary radiations. Despite sharing a similar habitus, they evolved various arboreal locomotor specializations (e.g., Berles et al., 2021; Berles et al., 2024; Buchanan‐Smith, 1991; Garber, 1980; Garber et al., 2012; Garber & Pruetz, 1995; Nadjafzadeh & Heymann, 2008; Norconk, 1990; Nyakatura & Heymann, 2010). All species are relatively small (120–700 g; Garber, 1992) and possess claw‐like nails for clinging to their supports (Garber, 1992; Garber et al., 2009; Porter & Garber, 2004). Most marmosets are found in heights of three to 15 meters (Buchanan‐Smith et al., 2000; Cunha et al., 2006; Yoneda, 1984), whereas most tamarin species occupy higher forest layers up to the canopy (e.g., Fleagle, 1999; Forbes, 1894; Yoneda, 1984). These preferences confront these primates with varying support properties that are reflected in species‐specific leaping modes and locomotor performance, typically divided into trunk‐to‐trunk leaping (Garber, 1991, 1992; Garber & Leigh, 2001; Youlatos & Gasc, 2001) and horizontal leaping (Jenkins, 1974; Nyakatura, 2019; Schapker et al., 2022; Silcox & Lopez‐Torres, 2017; Young & Shapiro, 2018). However, Berles et al. (2024) demonstrated that support preferences and locomotor performance can be highly variable and difficult to categorize, necessitating the acquisition of in‐depth behavioral profiles to understand the full extent of locomotor plasticity in these species.

Such locomotor diversity can be expected to be reflected in adaptations of the locomotor apparatus. For example, landing on large and inflexible supports (such as tree trunks) creates large stresses on the limb bones, while grasping and balancing are more important on small, flexible supports (Botton‐Divet & Nyakatura, 2021). In order to increase leaping distance, species preferentially performing trunk‐to‐trunk leaping possess, for example, elongated hind limb segments in comparison to horizontal leapers (e.g., Berles et al., 2024; Botton‐Divet & Nyakatura, 2021; Garber, 1992; Garber et al., 2005; Garber et al., 2012; Garber & Leigh, 2001). However, differences in osteological features are often nuanced (Botton‐Divet & Nyakatura, 2021) and sometimes contradict predictions based on biomechanical reasoning (Berles et al., 2024). It also remains unexplored if osteological differences translate into biomechanical differences that ultimately affect leaping performance. Features such as limb bone length and the topology of muscle attachments influence the leverage and effective contraction length of the muscles and thus the power and speed of the movements during climbing and leaping (Goslow & Hildebrand, 1995).

An important determinant of locomotor performance and particularly joint function is the muscle moment arm (MMA), which is the perpendicular distance from a muscle's line of action to the center of rotation of the joint the muscle is acting on (Hutchinson et al., 2005; Löffler et al., 2022; Michilsens et al., 2009; Murray et al., 2002; Visser et al., 1990). This distance in three‐dimensional (3D) space varies with joint angle, the MMA of a particular joint pose being referred to as the instantaneous MMA (e.g., Allen et al., 2021; Löffler et al., 2022). A long MMA—typically achieved by positioning of the muscle attachment sites far from the joint center of rotation—optimizes torque, while a short MMA benefits joint rotational velocity, albeit with relatively lower torque (Channon et al., 2010; Goslow & Hildebrand, 1995). To assess the effectiveness of MMAs at various joint angles, it is also important to consider the muscle strain, that is, the instantaneous length of a muscle divided by its resting length (determined by the muscle's length‐tension relationship; e.g., Lautenschlager, 2015). The optimal muscle strain for force generation plateaus near resting length (85%–115%) and animals appear to prefer optimal muscle strains, for example, during locomotion (Zwafing et al., 2021).

**FIGURE 1:**
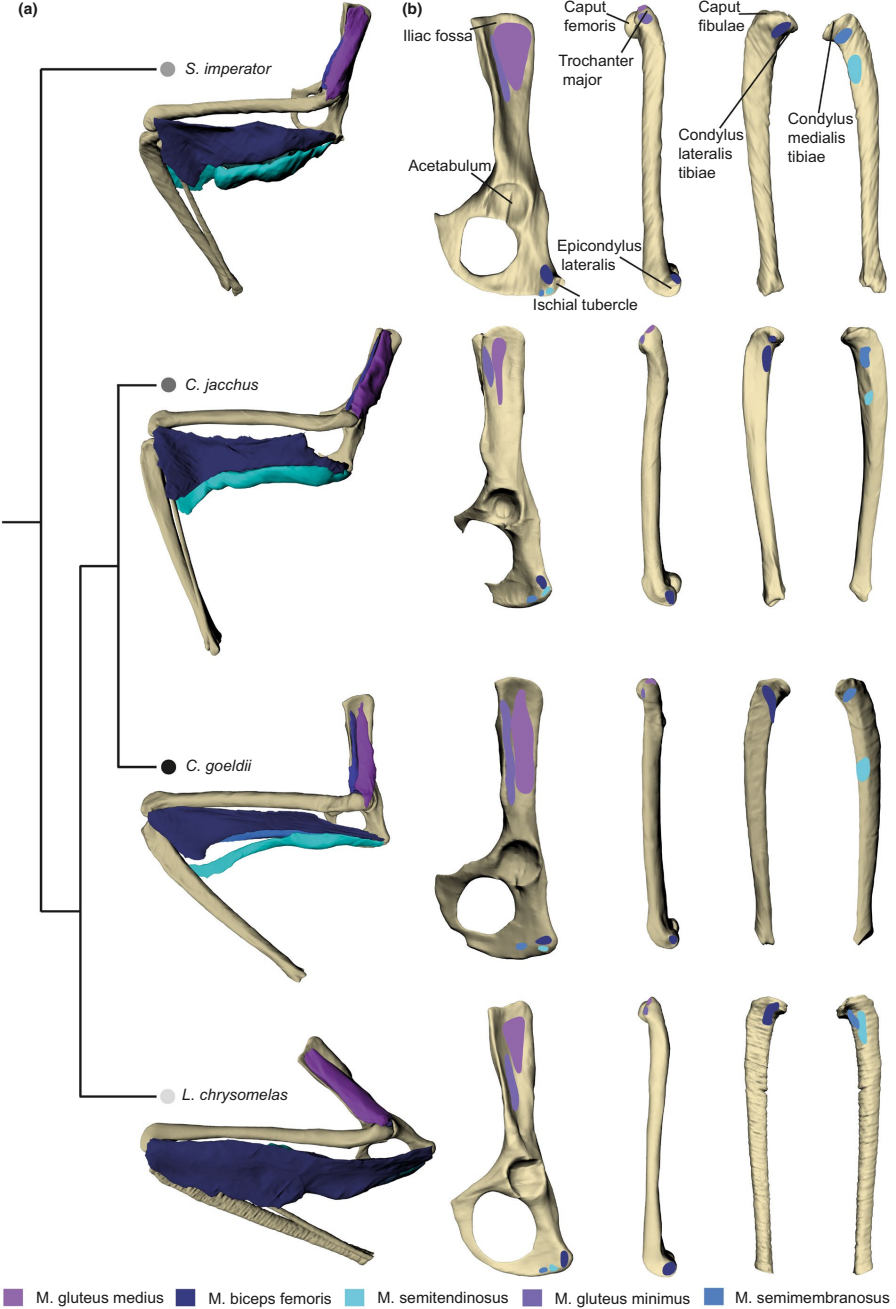
Phylogeny of the studied callitrichid species and topological information of the five studied hip extensor muscle of each analyzed specimen. (a) Phylogenetic relationships (topology based on Aristide et al. (2015) without branch length information) and 3D virtual models of the left extensor muscles, pelvis, femur, tibia, and fibula. (b) Muscle maps with origin on the pelvis and insertion on the limb long bones.

Here, we present a pilot study employing a computational modeling approach that compares instantaneous MMAs and muscle strains in the five most important hip extensors (gluteus medius and minimus, and the biarticular muscles biceps femoris, semimembranosus, and semitendinosus) between four different callitrichid species with different locomotor ecologies (Figure 1). *Callimico goeldii* prefers very low heights (Buchanan‐Smith, 1991; Izawa, 1975; Pook & Pook, 1981, 1982; Porter, 2004) and is a specialist in vertical leaping between trunks (Garber et al., 2005), covering distances of up to four meters in single leaps (Buchanan‐Smith, 1991; Buchanan‐Smith et al., 2000; Garber & Leigh, 2001; Pook & Pook, 1981). *Callithrix jacchus* resides predominantly in intermediate heights (Cunha et al., 2006; Davis, 2002; Fox et al., 2019; Rylands, 1981), where it clings vertically to trunks to feed on exudates and insects (Davis, 2002). *Saguinus imperator* usually stays at lower heights, but is specialized in horizontal leaping (Berles et al., 2024; Karantanis, 2010) and predominantly uses smaller, oblique supports for locomotion (Buchanan‐Smith, 1999). *Leontopithecus chrysomelas* prefers intermediate to large heights (Coimbra‐Filho & Mittermeier, 1973; de Monteiro Almeida Rocha et al., 2014; Fleagle, 1999), usually moving quadrupedally across level and oblique branches of small to medium diameter (Coimbra‐Filho & Magnanini, 1972; Fleagle, 1999; Karantanis, 2010; Stafford et al., 1996).

We expect large MMAs as a potential adaptation to produce high torques to be associated with trunk‐to‐trunk leaping, benefiting the takeoff from a resting position with crouched limbs. In turn, smaller MMAs and thus, higher rotational velocity might benefit species that are specialized in leaping out of a run‐up, which might be the case in horizontal leapers that chase through the canopy.

## 2 MATERIALS AND METHODS

### 2.1 Specimen sample and preparation

To base our 3D virtual models on actual anatomy, one cadaver of each species (the emperor tamarin *Saguinus imperator*, the Goeldi's marmoset *Callimico goeldii*, the common marmoset *Callithrix jacchus* and the golden‐headed lion tamarin *Leontopithecus chrysomelas*) was provided without the internal organs by the Royal Zoological Society of Antwerp, Belgium. Sex and age were only partly known, but all specimens appeared to be adult (the epiphyseal plates were closed in all four species; Table S1). We lack information on the endowment within the enclosure of these individuals. However, since muscle attachment sites exhibit limited adaptability to functional demands over an individual's lifetime (Anapol & Barry, 1996; Turcotte et al., 2020), we hypothesize that muscle topology—and consequently, the instantaneous MMAs—remains largely unaffected, if not entirely unchanged, by the maintenance of individuals in captivity (Rabey et al., 2015). After temperature‐controlled transport to the Humboldt‐Universität zu Berlin, Germany, the carcasses were thawed and measured in terms of head‐torso length, length of front and hind limbs, and eviscerated body mass (Table S1).

Preparation of the four specimens followed the protocol of Eigen and Nyakatura (2021). After removing the skin, the cadavers were fixed in position with cable ties and then fixed in a phosphate‐buffered 4% formaldehyde solution (Histofix, Carl Roth GmbH, Karlsruhe, Germany) for 3 days (Table S1). Subsequently, they were rinsed with water for 1 h and immersed in successive ethanol baths of increasing concentration (15%, 30%, 50%, 60%, and 70%) for 1 h each (Descamps et al., 2014; Metscher, 2009a, 2009b; Pauwels et al., 2013). To increase contrast in subsequent μCT scans, we stained the preparations with PTA (3% phosphotungstic acid in 70% ethanol; Koç et al., 2019) on a laboratory rocker for constant circulation for several days (Table S1).

### 2.2 Digitization and length measurements of bones and muscles

The specimens were digitized with a helical scan and a detector time of 0.8 s at the Museum für Naturkunde Berlin, Germany, using an YXLON FF35 CT (YXLON International GmbH, Hamburg, Germany). Bones and muscles were segmented using AMIRA (version 6.0.0., Thermo Fisher Scientific, Waltham, USA) and the 3D surface models jointly exported to Geomagic wrap (3D Systems 2017, Rock Hill, USA) for measuring the effective lengths of femur and pelvis, for determining muscle attachment sites (Figure 1), and for smoothing for visual representation.

### 2.3 Instantaneous muscle moment arms and muscle strains

MMA and muscle strains as well as the length of each muscle were determined in Autodesk Maya 2022 (Autodesk, San Rafael, United States). Reference position was defined with 90° angles at the hip and knee (this also defined the resting length of the hip extensor muscles; Figure S1a). The calculation of instantaneous MMA has already been described in detail by Löffler et al. (2022) and will only be briefly summarized here. We fitted a sphere into the femoral head, its center representing the approximated hip joint center of rotation. Each muscle's line of action was determined by connecting the centers of the origin and insertion with a straight line. We determined the MMA of each pose by using the ‘nearest point on curve’ function, which identifies the point along the muscle line of action that is closest to the joint center of rotation (Figure S1b). Instantaneous MMAs and muscle length (to calculate muscle strain) were measured for hip extension angles at intervals of 10° of rotation of the femur relative to the hip from 20° extension (i.e., strongly flexed) to 160° extension (Figure 3a; Thorpe et al., 1999; Williams et al., 2008). This was based on maximum in vivo hip range of motion from 26° to 158° in white‐handed gibbons when leaping from a low crouched position (Channon et al., 2010). Similar values appear reasonable for callitrichids, although detailed kinematic data for hip extension during leaping is not available. Following Williams et al. (2008), we always kept the knee, which was not the primary joint in focus, in a fixed, neutral position (90° extension angle) relative to the femur (reference pose). This was important for the bi‐articulate muscles which pass the hip and knee joints and for which knee flexion/extension also in part affects the instantaneous MMA relative to the hip joint. We considered optimal muscle strains (i.e., the muscle's length‐tension relationship) to fall between 85% and 115% sensu Lautenschlager (2015). To allow comparison of specimens of different size, we divided muscle and bone length as well as MMAs by body mass^0.33^ (cf. Allen et al., 2010). Given that a muscle, due to an oblique orientation, typically contributes to multiple axes of joint rotation in a six‐degree‐of‐freedom joint (e.g., flexion/extension, abduction/adduction, and long‐axis rotation), we projected all measurements of MMAs onto the flexion–extension plane—a parasagittal plane passing through the hip joint's center of rotation. Consequently, only the muscle's contribution to hip extension was evaluated.

We are well aware that musculoskeletal modeling can also include a variety of biases. As previously shown by Charles et al. (2016), a shift in the origin or insertion site of the muscles can have a large effect on instantaneous MMA. In our study, we used the center of the attachment points, and model setup was always performed by the same person (PB). At the beginning of our study, we performed a sensitivity analysis to test the effect of center of origin placement on the measurement of the instantaneous MMA. For this purpose, PB repeated this placement independently on five different days. We compared the variability between measurements to the interspecific variability by generating error bars in R Statistical Software (v4.3.1; R Core Team, 2024). We found only negligible variability in repeated measurements of the instantaneous MMA (Figure S2). Two further sensitivity analyses were performed to assess the robustness of the model. Firstly, to account for uncertainty in the body mass measurements, each body mass value was changed by adding or subtracting a fraction of its original value (+10%, +20%, −10%, −20%) and secondly, the origin of the biceps femoris was shifted along the bone as a percentage of pelvic length (+10%, +20%, −10%, −20%) to analyze the effects of these changes on the determined muscle parameters using *C. goeldii* as an example. Here, too, we found only negligible variability in the results (Figure S3). Based on these results, we determined that our data provide a good estimate of instantaneous MMA and muscle strains. Our data thus provide insight into the effectiveness of the muscles in performing movements in specific joint positions (cf. Charles et al., 2016).

## 3 RESULTS

**FIGURE 2:**
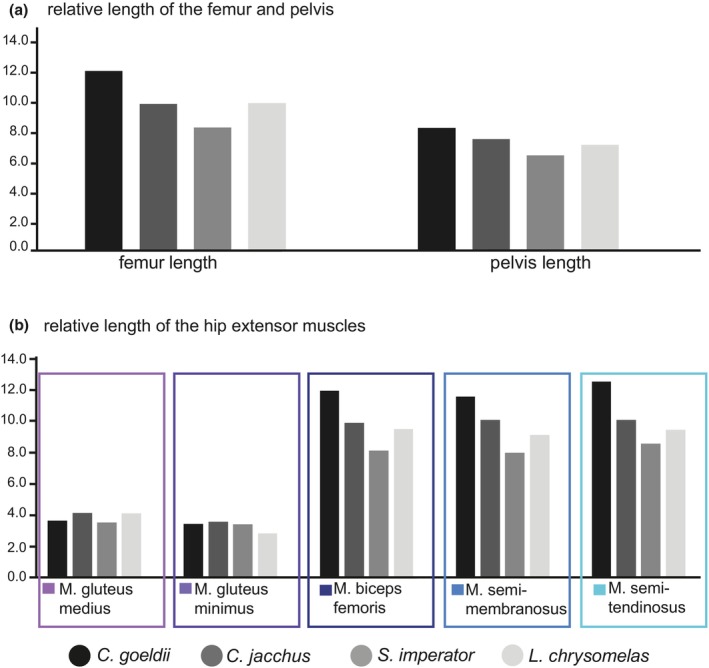
Bar plots for comparison of relative length measurements of skeletal elements and hip extensor muscles between the four studied callitrichid species. (a) Relative lengths of femur and pelvis (in relation to the individual's body mass^0.33^; cf. Allen et al., 2010). (b) Relative lengths of extensor muscles at our reference pose of the hip joint (90° hip extension, see Figure 3a, color code according to Figure 1).

**FIGURE 3:**
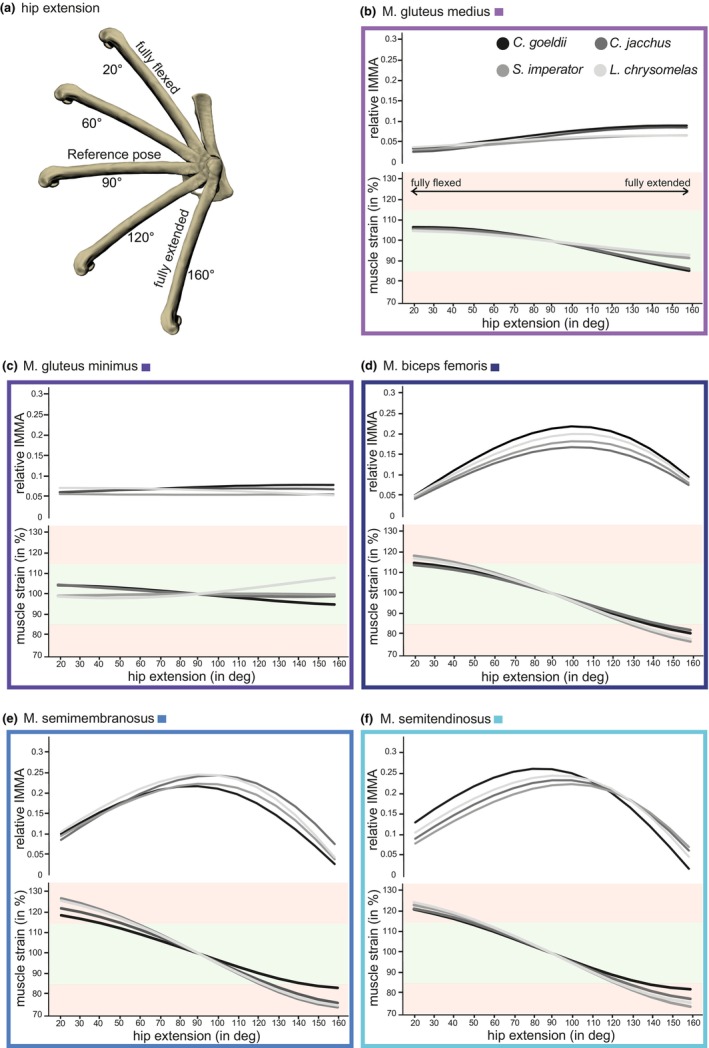
Virtual modeling of the 3D instantaneous muscle moment arms and muscle strains of the hip extensors projected onto the extension/flexion plane. (a) Illustration of the joint positions of the hip (femur in relation to pelvis) of *C. goeldii*. Femoral retraction in the hip was analyzed in 10° increments from 20° to 160°. (b–f) Relative instantaneous muscle moment arm (IMMA) and muscle strains of the hip extensors of the extension phase in the four studied callitrichid species. The black line represents the specialized trunk‐to‐trunk leaper, the different grey values depict horizontal leapers. Light green area: Optimal length‐tension relationship for force generation (85%–115%); Light red areas: Increasingly suboptimal length‐tension relationship with more limited potential for force generation.

The *C. goeldii* specimen had the relatively longest femur, pelvis, and extensor muscles of the hip joint (except for the two gluteii; Figure 2). This exception might be related to the gluteus origins covering almost the entire lateral surface of the iliac wing (i.e., a considerably larger area than in the other species), resulting in the center of attachment being closer to the hip joint. *C. goeldii* also displays the longest MMA for the gluteii and the biceps femoris and—from 20° to 100° hip joint extension—for the semitendinosus. As in all studied specimens, the MMA of the gluteii tends to increase with hip extension angle, where those of the biarticular muscles followed a bell‐shaped curve with largest values around the reference pose (90°). The muscle strains of its gluteii always lie within the optimum for force generation (which was the case for all species) and only fall outside for the other muscles close to the minimum and maximum hip extension angles (Figure 3b–f).

The *S. imperator* specimen had the relatively shortest skeletal elements and muscles in relative terms (Figure 2). Its muscles were always among those displaying the shortest MMAs, despite some centers of muscle attachments being located comparably far from the joint center of rotation (e.g., gluteii). *S. imperator* always showed the narrowest range of optimal muscle strains for biceps femoris, semimembranosus, and semitendinosus, although substantial differences between the studied specimens were only observed for the semimembranosus (Figure 3b–f).

*L. chrysomelas* and *C. jacchus* almost always had very similar and—compared to the others—intermediate values of relative bone and muscle lengths (Figure 2). They also mostly possessed intermediate MMA values; however, both had the largest MMAs for the semimembranosus, although the associated muscle attachment sites were not particularly distant from the hip joint. In *C. jacchus*, as in *C. goeldii*, the insertion of the semitendinousus is shifted toward the center of the tibial shaft (Figure 1b), but its MMA values are much smaller than those from *C. goeldii*, comparable to those of the other two species (Figure 3f). Also, the insertion of the semitendinosus of *S. imperator* is located closer to the center of the tibial shaft in comparison to *L. chrysomelas* (Figure 1b). Regarding the biarticular muscles of *C. jacchus*, the range of optimal strains was very similar to *C. goeldii*, whereas *L. chrysomelas* shared similar ranges with *S. imperator* (Figure 3d–f).

## 4 DISCUSSION

Due to the analyzed species' differently structured micro‐habitats (preference of vertical tree trunks vs. terminal branches in the canopy) and different leaping modes (vertical clinging and leaping vs. horizontal leaping, respectively; cf. Berles et al., 2021), we expected that the extensors of the hip reflect the resulting differences in the functional requirements (Löffler et al., 2022; Nyakatura et al., 2019). Although our computational modeling approach revealed many similarities between the studied specimens, there were also noticeable differences in our preliminary results, which partly met our expectations related to potential adaptations to differing locomotor specialization.

*C. goeldii*, a proficient trunk‐to‐trunk leaper (Garber et al., 2005), possessed the largest MMAs for most of the analyzed muscles and hip extension angles. This agrees with the assumption that large torques are necessary for powerful acceleration from a clinging position to a high takeoff velocity to overcome large leaping distances. The fact that semitendinosus MMAs only exceeded those of the other species during smaller extension angles might reflect the need to generate large torques while still clinging to the trunk in a crouched (i.e., almost fully flexed hip) position. Relatedly, the *C. goeldii* specimen having the relatively largest areas of origin of the two glutei might also reflect that these muscles experience large stresses during vertical leaping. Furthermore, the longer optimal length‐tension range for the *C. goeldii* specimen might be another optimization for force generation from a crouched position all the way to an almost fully extended leg position. This allows a higher muscle efficiency to be maintained during muscle tension (>100% of the resting length) and muscle compression (<100% of the resting length) (Lautenschlager, 2015; Voigt et al., 1995). It is known that *C. goeldii* has elongated hind limbs in comparison to close relatives: all hind limb long bones are long compared to other tamarin species (Garber et al., 2009; Garber & Leigh, 2001). Moreover, *C. jacchus* is also characterized by very long and slender limb bones in comparison to the other species of Callitrichidae (Casteleyn et al., 2012). The elongated limbs in combination with the longer optimal length‐tension range may favor a higher takeoff speed, allowing the possibility of covering greater horizontal distances, as has already been described for strepsirrhines specialized in vertical clinging and leaping (Demes et al., 1999; Garber & Leigh, 2001; Gebo, 2011). Secondly, it may benefit leaping distance from a crouched position by positioning the center of mass of the animal further away from the substrate, thus minimizing the effective flight distance (Channon et al., 2011).

In contrast, *Saguinus imperator* appears to be optimized for fast hip joint rotational movements with a slightly more constrained optimal range of motion when compared to the other specimens in our sample. Like *L. chrysomelas* and *C. jacchus*, it prefers horizontal leaps (Cunha et al., 2006; Karantanis, 2010). However, the fact that it typically leaps off from intermediate heights, using oblique (Buchanan‐Smith, 1999), and perhaps less flexible supports (supports tend to be of a larger diameter in lower forest strata in comparison to the canopy), might partly explain the overall smaller MMA values. *L. chrysomelas* and *C. jacchus*, facing thin and flexible supports in the lower layer and canopy (Coimbra‐Filho & Mittermeier, 1973; Cunha et al., 2006; de Monteiro Almeida Rocha et al., 2014; Fleagle, 1999), might rely on relatively larger MMAs to achieve similar takeoff speeds. *C. jacchus* and *C. goeldii* share a similar insertion of the semitendinosus relatively distant from the knee, but both differ in the corresponding hip joint MMA. This distant insertion might be explained by increasing the knee joint MMA, perhaps optimizing knee flexion torques for holding tightly to the support when clinging to the trunk (cf. Hildebrand & Goslow, 2001).

In conclusion, we here provide tentative evidence that computational modeling of MMAs and muscle strains can be a useful tool for understanding joint function and biomechanical adaptations beyond simple osteological measurements in Callitrichidae. While tamarins and marmosets share a very similar habitus, small‐scale differences in body proportions and muscle topology can result in changes in overall biomechanical performance that ultimately explain the observed locomotor diversity within this radiation of primates. On the other hand, retaining a rather generalized musculoskeletal system for hip extension with only moderate specializations appears to allow callitrichids to achieve locomotor plasticity in highly complex arboreal habitats (cf. Berles et al., 2024). More species/specimens are necessary to generalize the presented trends, and the inclusion of other functional parameters, such as the muscles' cross‐sectional area (cf. Channon et al., 2010), could further improve biomechanical inferences.

## AUTHOR CONTRIBUTIONS

PB and JAN conceived of the study. PB and JAN designed the methodology. PB collected the data. PB and JW analyzed the data. PB drafted the manuscript. All authors revised the manuscript and gave final approval for publication.

## FUNDING INFORMATION

This study was funded by the German Research Foundation DFG (NY 63/2–1) and a DFG large instrumentation grant (INST 276/851–1 FUGG).

## CONFLICT OF INTEREST STATEMENT

The authors declare that there is no conflict of interest.

## Supporting information


**Figure S1.** Illustration of muscle geometry using the virtual bone models of *C. goeldii*. (a) Black lines represent the hip extensor muscles’ line of action and were used to measure the muscles’ resting length at 90° hip extension. (b) Measurement of the instantaneous muscle moment arm at right angle to the muscle line of action. Blue and red lines: sphere fitted into the femoral head, its center representing the approximated hip joint center of rotation, green line: instantaneous muscle moment arm.
**Figure S2.** Sensitivity analysis of repeated locator placements for measurement of instantaneous muscle moment arms at three different hip extension angles. Relative (size‐corrected) instantaneous muscle moment arms on the y‐axis. Small grey circles: data points. Large black circles: mean values. Grey error bars: standard deviations. Note that the standard deviations were often very small and thus, the error bars are barely visible in these cases.
**Figure S3.** Sensitivity analysis of the instantaneous muscle moment arm (MMA) on *C. goeldii*. Top: Measurement of the MMA throughout the range of motion, while shifting the origin of the M. biceps femoris. Bottom: Measurement of the MMA throughout the range of motion, with results normalized by percentage changes of the reference weight. The line colors representing the percentage changes.
**Table S1.** Samples and staining protocol of the individuals with phosphotungstic acid (PTA).

## Data Availability

All data and R code necessary for the reproduction of the results are available on Figshare (https://doi.org/10.6084/m9.figshare.28882196).
