# Supplementary material for: Joint function in marmosets and tamarins: Insights from computational modeling of hip extensor muscles
Source: J Anat. 2025 May 15;247(5):1028–37. doi: 10.1111/joa.14268 (PMC12497566; doi:10.1111/joa.14268)
Supplement: Supplementary file 1 — Figure S1. Illustration of muscle geometry using the virtual bone models of C. goeldii. (a) Black lines represent the hip extensor muscles’ line of action and were used to measure the muscles’ resting length at 90° hip extension. (b) Measurement of the instantaneous muscle moment arm at right angle to the muscle line of action. Blue and red lines: sphere fitted into the femoral head, its center representing the approximated hip joint center of rotation, green line: instantaneous muscle moment arm. Figure S2. Sensitivity analysis of repeated locator placements for measurement of instantaneous muscle moment arms at three different hip extension angles. Relative (size‐corrected) instantaneous muscle moment arms on the y‐axis. Small grey circles: data points. Large black circles: mean values. Grey error bars: standard deviations. Note that the standard deviations were often very small and thus, the error bars are barely visible in these cases. Figure S3. Sensitivity analysis of the instantaneous muscle moment arm (MMA) on C. goeldii. Top: Measurement of the MMA throughout the range of motion, while shifting the origin of the M. biceps femoris. Bottom: Measurement of the MMA throughout the range of motion, with results normalized by percentage changes of the reference weight. The line colors representing the percentage changes. Table S1. Samples and staining protocol of the individuals with phosphotungstic acid (PTA). [file JOA-247-1028-s001.docx]

**- SUPPLEMENTARY MATERIAL -

Joint function in marmosets and tamarins: Insights from computational modelling of hip extensor muscles**

**Patricia Berles^1*^, Jan Wölfer^1^ and John. A. Nyakatura^1^**

*corresponding author: patricia.berles@hu-berlin.de
^1^: Comparative Zoology, Institute of Biology, Humboldt-University Berlin, Philippstraße 13, 10115 Berlin, Germany.

ORCID:

Patricia Berles https://orcid.org/0000-0002-8069-748X

Jan Wölfer https://orcid.org/0000-0001-8630-2461

John A. Nyakatura https://orcid.org/0000-0001-8088-8684

**Content:**

| Table S1 | Samples and staining protocol of the individuals with PTA. | 2 |
| --- | --- | --- |
| Fig. S1 | Illustration of muscle geometry using the virtual bone models of *C. goeldii* | 3 |
| Fig. S2 | Sensitivity analysis of repeated locator placements for measurement of instantaneous muscle moment arms at three different hip extension angles | 4 |
| Fig. S3 | Sensitivity analysis of the instantaneous muscle moment arm (MMA) on *C. goeldii* | 5 |

**Table S1**. Samples and staining protocol of the individuals with phosphotungstic acid (PTA).

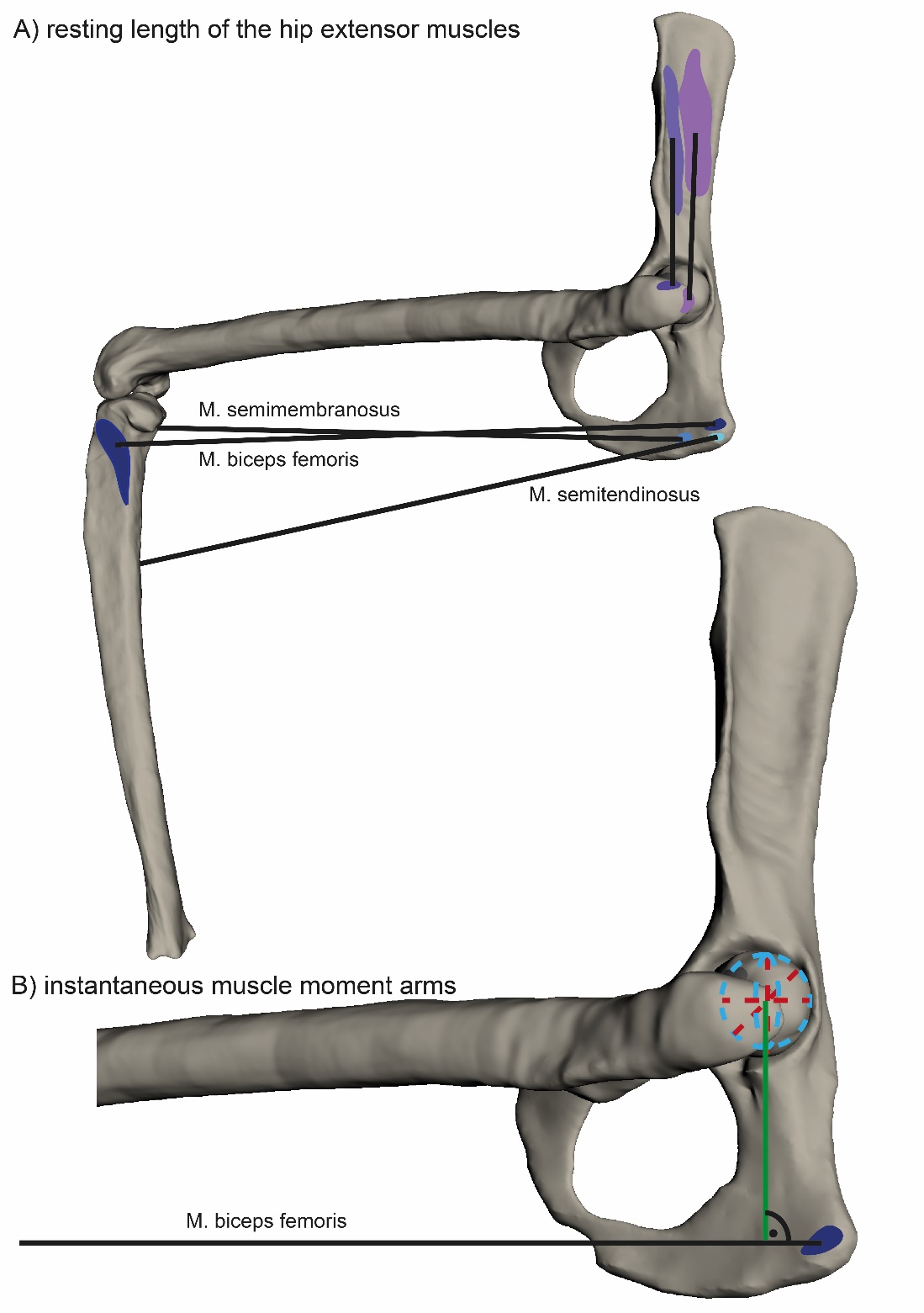


**Fig. S1** Illustration of muscle geometry using the virtual bone models of C. goeldii. A) Black lines represent the hip extensor muscles’ line of action and were used to measure the muscles’ resting length at 90° hip extension. B) Measurement of the instantaneous muscle moment arm at right angle to the muscle line of action. Blue and red lines: sphere fitted into the femoral head, its centre representing the approximated hip joint centre of rotation, green line: instantaneous muscle moment arm


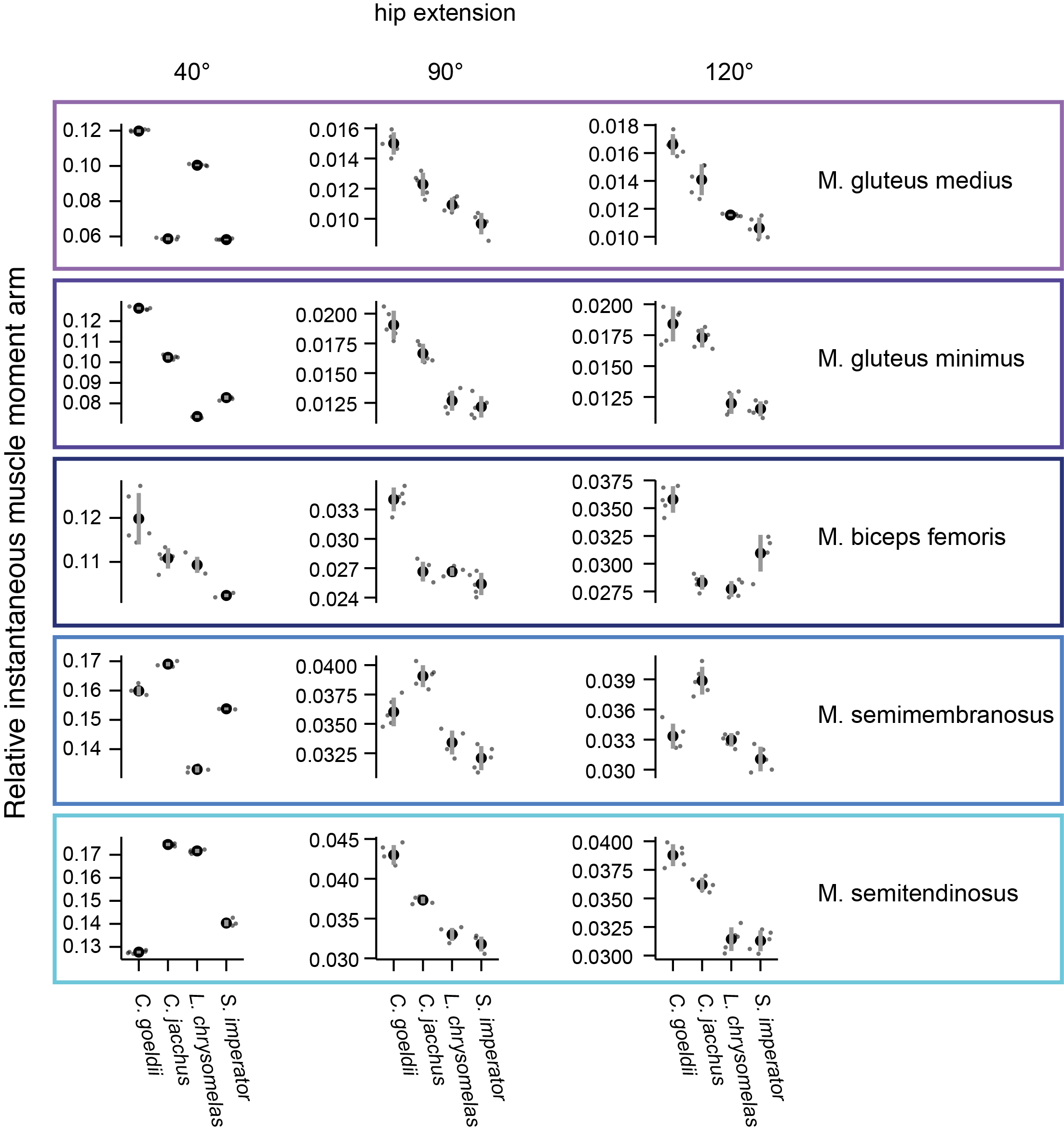


**Fig. S2** Sensitivity analysis of repeated locator placements for measurement of instantaneous muscle moment arms at three different hip extension angles. Relative (size-corrected) instantaneous muscle moment arms on the y-axis. Small grey circles: data points. Large black cricles: mean values. Grey error bars: standard deviations. Note that the standard deviations were often very small and thus, the error bars are barely visible in these cases


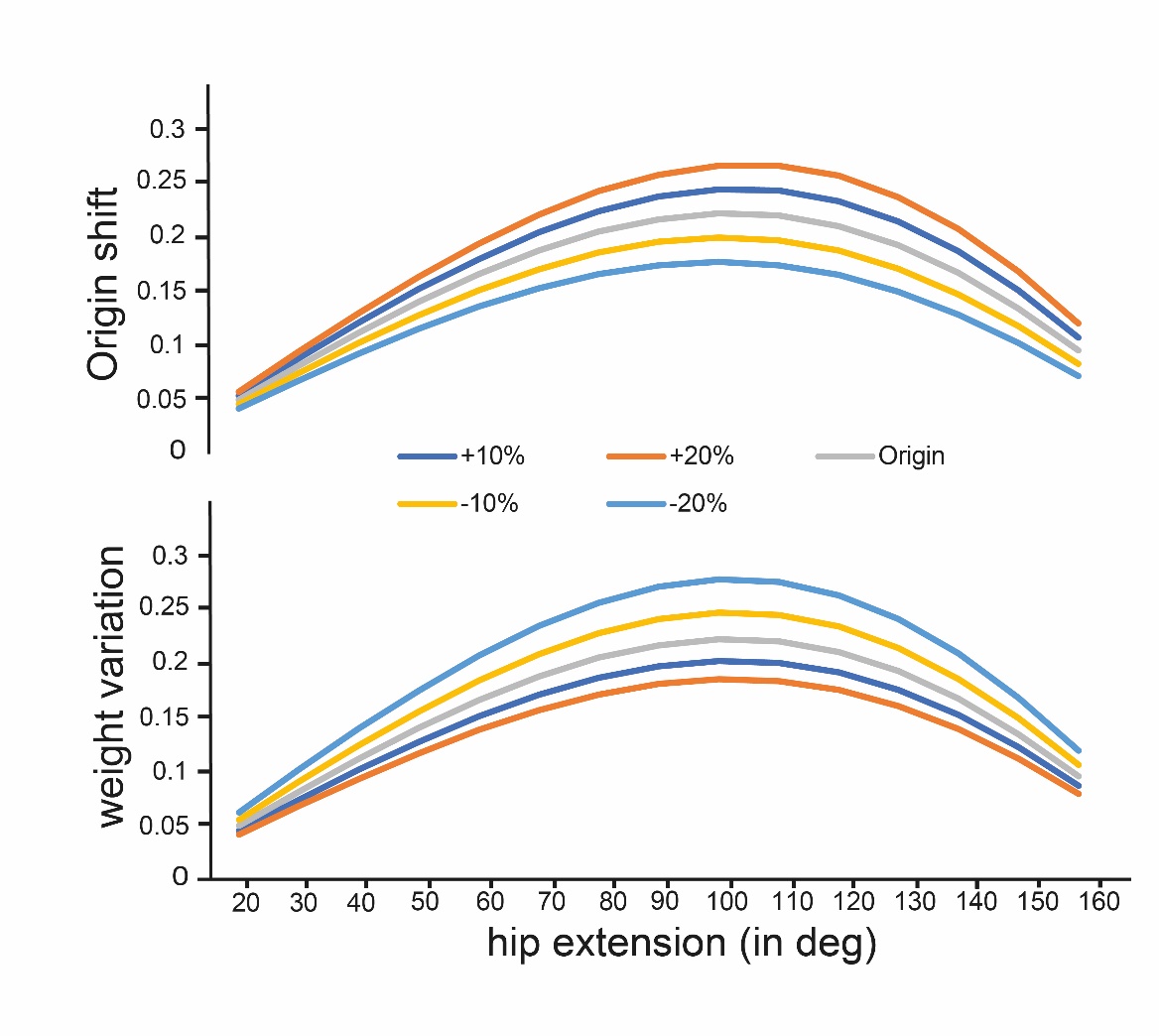


**Fig. S3** Sensitivity analysis of the instantaneous muscle moment arm (MMA) on *C. goeldii*. Top: Measurement of the MMA throughout the range of motion, while shifting the origin of the M. biceps femoris. Bottom: Measurement of the MMA throughout the range of motion, with results normalized by percentage changes of the reference weight. The line colors representing the percentage changes.
